# Progress and Challenges of Additive Manufacturing of Tungsten and Alloys as Plasma-Facing Materials

**DOI:** 10.3390/ma17092104

**Published:** 2024-04-29

**Authors:** Logan Howard, Gabriel D. Parker, Xiao-Ying Yu

**Affiliations:** 1 Materials Science and Technology Division, Oak Ridge National Laboratory, Oak Ridge, TN 37830, USA; 2 The Bredesen Center, 310 Ferris Hall 1508 Middle Dr, Knoxville, TN 37996, USA

**Keywords:** additive manufacturing (AM), laser powder bed fusion (LPBF), electron beam melting (EBM), direct energy deposition (DED), tungsten (W), irradiation effects

## Abstract

Tungsten (W) and W alloys are considered as primary candidates for plasma-facing components (PFCs) that must perform in severe environments in terms of temperature, neutron fluxes, plasma effects, and irradiation bombardment. These materials are notoriously difficult to produce using additive manufacturing (AM) methods due to issues inherent to these techniques. The progress on applying AM techniques to W-based PFC applications is reviewed and the technical issues in selected manufacturing methods are discussed in this review. Specifically, we focus on the recent development and applications of laser powder bed fusion (LPBF), electron beam melting (EBM), and direct energy deposition (DED) in W materials due to their abilities to preserve the properties of W as potential PFCs. Additionally, the existing literature on irradiation effects on W and W alloys is surveyed, with possible solutions to those issues therein addressed. Finally, the gaps in possible future research on additively manufactured W are identified and outlined.

## 1. Introduction

Nuclear fusion presents an extremely hostile environment subject to exposure of severe thermal loads, high-neutron-energy bombardments, and neutron fluences. The tokamak reactor reports ≥1026 n/m^2^ when operating in a steady state, at high fluxes, and under low-energy hydrogen (H) and helium (He) plasma irradiations [1,2,3,4]. Such aspects impose significant restrictions on the design of plasma-facing components (PFCs) and plasma-facing materials (PFMs). Therefore, improving the peak conditions of PFMs increases the performance of PFCs, enabling more freedom in future fusion reactors [1,5]. To address the technical challenges in PFMs for fusion devices, materials must be capable of operating in a fusion reactor environment for an extended time while maintaining those desirable traits.

Tungsten (W) and W alloys have excited increased interest as a potential PFM candidate for enduring such extreme conditions [1,6]. W is noteworthy as a potential PFM for both the International Thermonuclear Experimental Reactor (ITER) and the demonstration power plant (DEMO), proposed for divertor applications [7]. W is deemed as a suitable PFM candidate due to its sputtering behaviors, having a high threshold energy for sputtering (i.e., ~200 eV for D) and a low sputtering yield [2,3,8,9]. It also boasts a high melting point, high thermal conductivity, good neutron irradiation resistance, fast diffusion of H, and low retention. W does not form hydrides or co-deposits with tritium (T) [2,3,8,9,10]. W also has the advantage of having a low retention of H isotopes, deuterium (D), and T, which serves to elevate it above other materials [3,6]. Furthermore, W alloys and composites garner attention for consideration as PFMs, playing a role as heat sinks [3,11,12,13].

### Challenges in Manufacturing Tungsten as a PFM

There are challenges in adopting W and W alloys as a PFM. While W has advantages for applications as PFCs, such as in the first wall (the inner reactor wall) and divertor, research and development are needed to enable the ease of manufacturing W in PFM applications. W is a notoriously challenging material to handle, with a dominant failure mechanism being intergranular fracture due to impurities located at the fracturing grain boundaries [14,15,16]. The phase transition of W depends on the environment, with a high pressure inducing a change from a body-centered cubic (BCC) to hexagonal close-packed (HCP) structure (see Figure S1). At lower pressures, the BCC structure dominates and ensures ductile to brittle transition temperature (DBTT) behavior. High melting temperatures make traditional methods such as casting unfeasible, initiating hot crack formation in the mold and forming porosity defects in the part. Its high DBTT limits room-temperature workability due to its brittle nature at room temperatures, which limits the performance of manufacturing methods [17,18].

The DBTT of W is associated with its crack nucleation and propagation. The DBTT, which is 300 °C–400 °C, is inevitably encountered when processing at high temperatures during cooling [19,20,21,22]. The alloy composition may change the DBTT of W, widening the range from around −15 up to 450 °C [23]. When this occurs, thermal stresses imparted into the material during processing may lead to microcrack development [19,24,25]. This challenge is made worse by the dependence of cracking and embrittlement behaviors on the interstitial impurity content due to the sensitivity of the grain boundaries of W towards impurities [19,26]. A notable example is the role that oxygen (O) impurities play in altering the behavior of W, with several studies focusing on the aggregation of W oxide phases during solidification that causes weak grain boundary cohesion and crack formation [24,26,27,28,29]. This behavior includes the formation of a metastable β-W phase which adopts an A15 crystal structure. Several studies have hypothesized that the introduction of trace elements, such as zirconium (Zr) and titanium (Ti), might react with the interstitial O to create stable nanoparticles of various oxides and strengthen the grain boundaries of W [30]. However, since cracks were not eliminated in O low environments, these impurities have been shown to not be the only factor [19].

**Figure 1:**
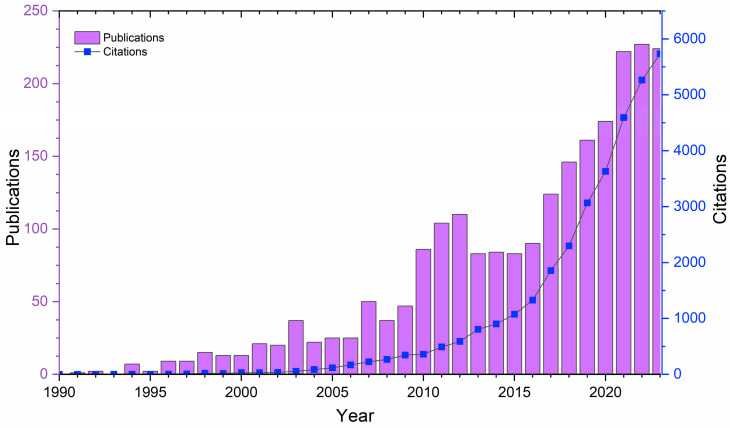
The number of publications on advanced manufacturing of W and W alloys. Adapted from data from Web of Science database [32].

Due to these influences, the traditional manufacturing of W has been plagued by short tool lives and chipping, given its brittleness [18,31]. To this end, where the limiting factors of these traditional manufacturing methods would impact the performance of W and W alloys as PFCs, the prospect of advanced manufacturing, specifically additive manufacturing (AM), has assumed a significant role. AM could provide a solution for W-based PFCs as it can offer a more successful and modern manufacturing approach for W. Substantial progress has been achieved in the development of AM and its applications to metals since the 1990s. Specifically, the number of publications regarding W has exponentially increased since 2008, as shown in Figure 1.

AM is a manufacturing process in which sequential layers of material are deposited according to specified computer-controlled parameters. AM offers advantages when compared with traditional manufacturing, especially when complex or arbitrary shapes are needed for increased geometrical design freedom and the ability to rapidly prototype components, as seen in Figure S2 [3,33]. AM also provides control of the microstructure and mechanical properties of W materials as a function of alloying elements and impurities, thermomechanical treatment, and production history [8]. W alloys play an important role in the performance of the final device made by AM, including the microstructure, mechanical properties, and influence of processing parameters on the as-manufactured materials.

**Figure 2:**
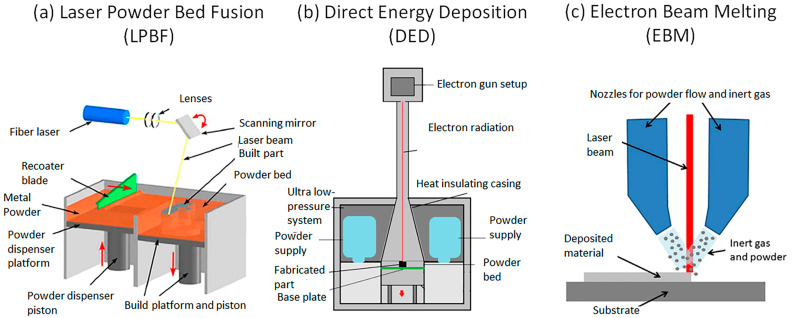
Overview of advanced manufacturing methods used for W and W alloys, showing a schematic of LBPF printing apparatus (**a**) [45], DED apparatus (**b**) [46], and EBM apparatus (**c**) [47]. Reprinted from Refs. [45,46,47]. with permission; Copyright Elsevier 2021, Woodhead 2020, and Woodhead 2021.

It is important to note that, across a wide range of studies, W is regarded as a challenging material for AM [3,34,35,36]. Several AM technologies are being considered to prepare W-based PFCs for fusion applications that have progressed in recent years [6]. Several recent reviews [26,37,38,39,40] cover the types of AM that have been applied to W. This review differs from others in that it intends to unify multiple manufacturing techniques and the resulting properties in the AM of W materials. It also seeks to highlight the irradiation effects that such materials will be exposed to. As shown in Figure 2, LPBF, DED, and EBM are three AM techniques that can facilitate the manufacture of parts from a wide variety of metals without the need for a binder phase [3]. They were chosen due to their promise in manufacturing W parts for PFCs. Other AM techniques exist for metals. However, they might not be suited for PFCs or have weaknesses that downplay their utilization for W. Binder jet printing is an example due to its reliance on the matrix in which W is sintered [41]. Metal material jetting is also deemed as unsuitable for fusion applications [38]. Studies will also use specific AM techniques as a proof of concept when analyzing a specific behavior [42,43,44]. LPBF, DED, and EBM are the most common techniques due to their suitability for producing W ideal for fusion purposes in ideal conditions.

The time range of this review extends from 1986 to January 2024. The focus is the development and application of the three representative AM techniques with an emphasis on W for fusion applications. We first give an overview of the LPBF, DED, and EBM techniques and discuss the specific issues and mitigating strategies associated with each technique. Differentiating mechanical and thermal properties are discussed. Also, an overview of irradiation effects is provided, although little information is available on AM-manufactured W. The environmental conditions for PFC survival and maintenance are discussed, along with the challenges associated with the utilization of W. Furthermore, recent relevant applications of machine learning (ML) in W AM are highlighted. Finally, challenges in unifying the previously mentioned fields are broached. Recommendations for future directions in W AM are proposed at the end.

## 2. LPBF, DED, and EBM Techniques for AM of W

### 2.1. Raw Material Characteristics

**Figure 3:**
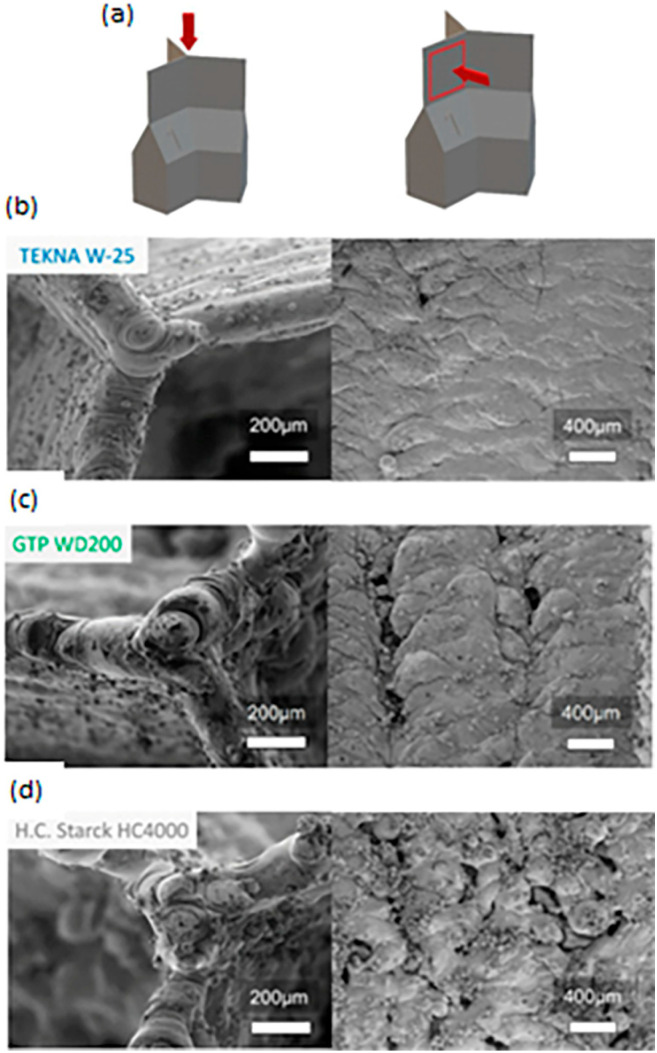
(**a**–**d**) Three LPBF thin-walled parts built with differing raw materials. Reprinted from Ref. [6] with permission; Copyright Elsevier 2022.

The raw powder characteristics of W influence the final W construct quality during the AM process [6]. These raw powder characteristics are attributed to the quality of the consolidated W part in powder-bed-based processes similar to traditionally manufactured materials [6]. Raw material purity is a chief concern regarding additively manufactured W, as impurities lead to the segregation of grain boundaries, a main cause of intergranular cracking [14,15,16]. Particle morphology, particle size distribution (PSD), and powder density are other factors. The desired particle morphology is spherical. PSD affects the powder flowability and the powder density within the powder bed during processing [6,48]. Recent results have shown that polyhedral W powder produced balling, large defects, and pores. Continuous tracks were present when using spherical powder. Microcracks and porosity occurred too [35]. Densification can be improved through the spheroidization of the raw powder [49]. Figure 3 shows the strong influence of the powder quality on the quality of the product. Figure 3b has better results compared to the Figure 3c (partly spheroidized) and Figure 3d (polygonal) powders in terms of porosity and surface quality. The thin-walled test specifically was shown to display distinct differences in terms of surface quality and porosity based on the raw powder utilized, with spherical powder providing better results [6]. Another study determined that spheroidized W powders are best suited for LPBF processing [50]. A list of the raw material suppliers can be found in Table S1.

Information on flowability and powder density is crucial for obtaining uniform layers during the manufacturing process, with the characteristics of the desired raw material sometimes differing between the processes in question. Powder characteristics that are secondary in influence include the chemical composition and optical properties of the powder, which affect the laser absorptivity [6]. Each of these characteristics influences the final quality and properties of materials manufactured using the LPBF process [6].

### 2.2. LPBF

LPBF was initially developed in an attempt to shorten prototyping timeframes in 1986 [33]. During LPBF, a powder is deposited layer-by-layer, where a single layer can range in thickness from 25 µm to 150 µm. Then, desired portions of each layer are selectively sintered via a laser beam. This process is generally performed in an argon (Ar) environment with varying O levels (i.e., 10–300 ppm) [26,51]. LPBF allows for thin-walled W parts to be manufactured, a traditional problem of some manufacturing techniques. Work has already been conducted to create complex W structures via LPBF, demonstrating that it is possible to apply AM techniques to pure W and W alloys [6]. There are a few critical parameters for LPBF that will affect the quality of the parts. These include the power of the sintering laser (laser power), laser scan speed spattered, beam diameter, layer thickness, and separation between one laser and the consecutive laser (hatch spacing) [26,52,53]. These parameters will influence the volumetric energy density [54,55].

Another crucial factor is the proportion of laser power that is imparted into the powder layer and laser absorptivity. One of the most popular parameters that has been studied for LPBF is the effect of these laser exposure parameters on the material quality of W [3,27,34,35,36,56,57,58,59]. LPBF, when compared to both DED and EBM, has a much smaller beam diameter, resulting in a stronger temperature gradient and higher cooling rate (see Table S2).

Table S2 summarizes the operating ranges of three methods, namely LPBF, DED, and EBM. The beam diameter used for LPBF is significantly smaller compared to that for DED and EBM, both of which have comparable beam sizes. A smaller beam size results in an increased temperature gradient while also experiencing a faster cooling rate. A higher temperature gradient allows for the use of high-melting-point materials in the AM process, such as W. The cooling rates show that the LPBF method is ~1000× faster than DED and EBM, which enhances the quality of the manufactured device by lowering the thermal stress and reducing the possibilities of cracking or pore defects. The particle size for LPBF is an order of magnitude smaller than that for the other methods, which also helps to reduce cracking and other defects commonly experienced using these three AM methods. These methods have benefits depending on the materials and the process chosen. Each technique proves to be useful when working with challenging materials such as W.

During laser scanning and melting, powder particles near the laser diameter are blown up [60]. Some powder particles are dragged to regions of the powder bed, leading to a clearing of particles around the track where the laser has melted across the bed, a phenomenon known as denudation [61]. Both spattering and denudation are common features of LPBF processes, which can lead to processing defects such as cracking and pores.

### 2.3. EBM

The EBM process is similar to LPBF. An electron beam as the heat source is moved across a powder bed to selectively melt some feedstock in sequential layers, as during LPBF. This process is often performed in a vacuum to maintain the quality of the electron beam, desirable for higher-energy applications [26]. EBM is often performed at raised temperatures, with the beam being used to raise the substrate temperature and the W materials being heated between 1000 and 1400 °C [62]. The beam can be defocused rapidly and swiped across the powder bed in between layers [44]. This enables the partial sintering of the material and permits the negative charge of the beam to be wicked away. Otherwise, the particles can be negatively charged and repulsed from one another. When compared to similar parts made using LPBF, materials made by EBM usually have lower thermal stress levels due to the heat produced in the processing [62].

Another advantage of EBM is that the electron beam can be manipulated over the entire build area or powder bed. This results in shorter-range control over the thermal conditions, better manipulation of the microstructure, and reduced thermal stresses [63,64,65]. EBM has been claimed to manufacture pure W without cracking, and the equipment for EBM is costly. With an unstable beam diameter and surface roughness, the dimensional accuracy of the parts is uncertain [66]. Varying states of EBM-fabricated W can be seen in Figure S3.

### 2.4. DED

DED ranks second after LPBF in terms of popularity [67]. Early DED used a laser-based system for large Ti parts. Metal powder is fed into the melt pool of a laser head to produce parts in DED. Later efforts expanded these technologies to broader applications [67]. DED differs from LPBF in that the feedstock is not rolled layer-by-layer into the bed but deposited by a head as a powder or wire. After one layer has been deposited and consolidated, the powder nozzles may move up to begin depositing another. DED is usually conducted under an atmosphere of Ar [68]. The feedstock for DED is metal powder or wire, with powders resulting in a lower deposition efficiency compared with metal wires. During consolidation, only a portion of the total powder will be melted and bonded to the substrate [46]. The DED process creates structures with good metallurgical bonds in between layers and from the part to the substrate [59,69]. Due to the extra freedom that comes with the additional design parameters that DED offers, it is suitable for the quick manufacturing of sizable parts with complex shapes.

DED has additional design parameters. Concerning the powder feed rate, the powder is deposited onto the part rather than a layer being created uniformly via a roller, unlike LPBF. Since the powder comes from some input, multiple hoppers can facilitate the inclusion of powders/powder compositions. This is a tremendous advantage that enables the manufacture of composite materials and the specific control of intra-part composition. DED can be used for repair because it can deposit material onto failed areas. This feature is beneficial for overcoming the challenge of joining dissimilar materials, such as W and structural steels [26].

## 3. Characterization, Properties, and Performance Evaluation of AM W and Alloys

### 3.1. Microstructures of LPBF

The ability of LPBF to selectively melt metals makes it an optimal choice for fabricating W and W alloys. W exhibits severe cracking because of a high DBTT because it cannot withstand the thermal stress caused by the rapid and repetitive local heating, sintering, solidification, and cooling cycles that LPBF imposes on materials. Also, geometric distortions and altered mechanical properties can occur during LPBF [19,70,71]. Even with extremely high densities (>98%), microcracks were not avoidable [3,34,58,72,73]. A solid, crack-free W cube was produced with a femtosecond fiber laser at a 1 MHz frequency [74]. However, a reduced tensile strength and hardness were found due to pores within the grain structure and discontinuities.

Two main types of cracks have been observed. In longitudinal cracking, the crack propagates parallel with the direction of the laser scan. In branched/transverse cracks, the crack propagates perpendicular to the direction of the laser scan [19,57,72,75]. Cracks also occur more along high-angle grain boundaries [19], with the regions next to cracks having lower kernel average misorientation (KAM) values when compared to those regions without cracks [76]. Electron backscatter diffraction (EBSD) studies on cross-sections of printed W samples have shown that thermal stress plays a role in the cracking behavior of LPBF-manufactured W [27,29,35,36,57,75,77]. The density of high-angle grain boundaries and cracks were found to be correlated, giving credence to the behavior where these cracks will relieve intergranular stresses caused by the LPBF process [27,29,57,77]. Oxygen impurities often present in the raw powder feedstock and develop W oxide phases during the solidification process, leaving pores of gas trapped within the grain boundaries. As the material continues to cool and solidify, these pores impart significant stresses on the material, leading to microcracking [56,78]. One hypothesis is that, at elevated temperatures, oxygen will readily react with W, which then segregates upon cooling to grain boundaries where it stimulates intergranular cracking [24]. Fine-grained W is more resistant to intergranular crack growth due to the more difficult routes for crack propagation [79]. In accordance with oxygen impurities altering the behavior of W, several studies have attributed the aggregation of W oxide phases during solidification as causing weak grain boundary cohesion and as the cause of crack formation [26,27,28].

If the main cause of cracking for W materials in LPBF is the thermal stresses caused by the heating loads imparted by the laser onto the part, then strategic alterations of the manufacturing may alleviate such stresses and reduce cracking. Strategies to suppress cracking in W parts manufactured using LPBF include alloying, remelting, substrate heating, and the adjustment of scanning parameters, raw material optimization, and post treatments (e.g., hot isostatic pressing).

#### 3.1.1. Alloying

**Figure 4:**
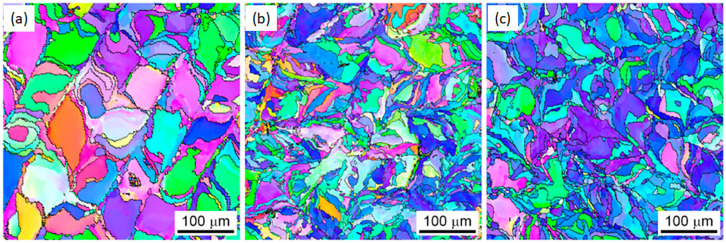
EBSD images of three Ta-alloyed LPBF W samples. Normal direction inverse pole figure (IPF) maps of cross-sections from pure W (**a**), W-5 wt.% Ta (**b**), and W-10 wt.% (**c**) are shown, respectively. Grains become more refined between (**a**,**b**), and a smaller change is noticed in (**c**). Reprinted from Ref. [34] with permission; Copyright Elsevier 2018.

A common approach to suppress cracking is by doping W with other stable elements. W alloys with elements such as tantalum (Ta) and Molybdenum (Mo) were shown to suppress cracking. [80,81]. The addition of 5 wt.% (Ta) powder to W powder decreased the grain size. However, increasing the amount of Ta to 10 wt.% produced no further decrease in grain size (Figure 4) [34].

Grain refinement was reported as a possible reduction in the cracking phenomenon that plagues W materials in LPBF. One explanation is that Ta offers a higher electron affinity to O than W. The formation of Gibbs free energy for Ta_2_O_5_ is −1904 kJ/mol, compared to a value of 761.5 kJ/mol for WO_3_ [66]. This causes Ta to attract more of the O impurities in the W powder, mitigating any segregation along the W grain boundaries [56,66]. Other studies found that W—Ta alloys form a W5Ta solid solution phase, which lowered the W melting point with an 80% reduction in cracking [56,77]. These reductions are thought to occur through nanoscale pores left by segregated or evaporated oxide phases at grain boundaries. Imposing distributions of nanopores through specific structures were suggested to trap these nanopores within grains and cell walls, instead of being concentrated at grain boundaries. Microstructure changes may accommodate the O impurities and allow for plastic deformation to alleviate residual stresses from these nanopores formed during manufacturing [56,77].

Creating alloys may promote a near complete reduction in cracking if combined with substrate heating. However, more comprehensive studies are warranted. Other molecular alloys (i.e., ZrC) were shown to reduce microcracking through a similar mechanism, in which Ta aids in grain refinement via the formation of ZrO_2_ [77]. Niobium (Nb) was also used to produce similar effects, chosen for its homogenous mixing with W and ductility [77]. Mo was studied but found to contain defects above the critical size for manufactured part failure [78].

#### 3.1.2. Parameter Alterations

The optimization of the build parameters, or build environment, offers a solution for mitigating concerns about the build quality. If the energy density of the as-built part is too low, it can lead to interrupted melting tracks, implying the formation of porosity. Conversely, if the energy density is too high, it can result in deep melting tracks with keyhole porosity formation [3]. Such behavior implies a moderate regime, in which desired properties may be reliably created. The creation of crack-free samples was reported by utilizing a nitrogen (N_2_) atmosphere to reduce the oxide levels at grain boundaries, increasing the boundary cohesion [82].

Another notable example is a phenomenon of the spherical agglomeration on the surface of AM parts known as balling [83]. On an initial scan, melted W droplets solidify into spherical geometries rather than forming a flat layer [49]. A reduction in the scan speed focuses the input energy and results in the formation of the molten track rather than balling [84]. These tracks alleviate the balling phenomena [18] and increase material densities [49]. Densification can be improved by overcoming the balling effect by improving the raw materials and optimizing the laser parameters (e.g., laser power and scanning speed) [49]. However, parameters have limits to their range of influence, for instance, the relative density of parts plateauing with a specific energy input [85].

As discussed earlier, spheroidal powders can increase laser absorptivity, enhance track formation, and improve densification [18]. Parameter optimization and raw material quality mutually improve the manufactured part properties in AM techniques. A chemical composition analysis of raw powder investigates the presence of crack nucleating impurities. Analyzers of C, N, O, and H are used to identify crack risk impurities. Between-layer rotations can help to reduce cracking for W parts [48]. For instance, when rotating between each layer (around 67°), grain orientation and shape were randomized, which reduced cracking. Non-rotated, non-random oriented grains resulted in a ladder-shaped structure. This process induces cracks, since these ladder-shaped grains are liable to become active sites. Most reports used rotation between layers (most often 67, 45, or 90°) to reduce the alignment of scans in the same direction, though this did cause texture in the (111) plane [86].

#### 3.1.3. Remelting and Substrate Heating

Remelting is the process of rescanning a track more than once before starting a new layer of powder. It helps to reduce grain size, improve surface roughness [59], and alleviate balling through the remelting of the globular droplets [49]. It also reduces longitudinal cracks and eliminates the tendency for grains to grow in columns due to the laser tracks, which can be observed in Figure S4. However, it was found to be insufficient for the complete elimination of cracks solely [26,59]. Substrate heating is another strategy used to suppress cracking in LPBF-prepared W components [3]. The residual stresses imparted by the laser are alleviated by reducing the temperature gradient. Efforts were attempted to combat embrittlement through the manipulation of the DBTT [3,19]. The underlying theory is that if the substrate is preheated above the DBTT of W, screw dislocations have enough mobility to accommodate the strain induced by the temperature gradient during the remelting/cooling cycles. Impurities can shift the DBTT of W from 623 K to 823 K by increasing the O content from 10 ppm to 50 ppm during substrate heating [87]. It was demonstrated that heating between 80 and 100 °C was not enough to eliminate cracking. However, cracking was significantly reduced with a density of above 99% at 1000 °C [3,6]. LPBF was also used with substrate preheating. Reduced cracking was obtained in LPBF-prepared W by a large margin when increasing the temperature from 200 °C to 1000 °C. However, the complete elimination of cracking is yet to be achieved [3].

### 3.2. Microstructures of EBM

Research has been conducted to study columnar grain structures that align themselves parallel to the build direction in EBM-prepared W [26]. Interlayer rotation is a strategy that might control this phenomenon [88]. EBM-prepared metals, such as W, are known to have texturing, with little significant link to its effect on processing [89]. EBM has shown a range of successful prints of W materials. One of the often erred parameters is transmitting too much energy to the material, either causing excessive fusion and swelling or expansion due to under-sintering [90].

Fusion happens in a characteristic disturbed melt pool due to excessive energy input or particle attachment [90]. The porosity of EBM-prepared W was characterized. The resulting properties were related to the theoretical density of W. Insufficient fusion occurred with relative densities of <70% in W that had conglomeration in the melt section. For relative densities between 70% and 90%, significant interconnected porosity was observed. Proper fusion can be achieved in areas where above 90% of density is reached [66]. Microcracking was observed despite a 99.5% density [90].

W materials with comparable densities were prepared using LPBF and EBM. However, both processes have common issues in cracking due to alloy selections, parameter alterations, and the substrate/build temperature for mitigating porosities and defects [44,91]. Cracks are anticipated to occur along grain boundaries as a result of deformation via the oxide presence in EBM-prepared materials [24]. Thus, the thermal gradient during cooling, either in LPBF or EBM, can drop W to below its DBTT and cause thermal stresses to impose cracking along low-strength grain boundaries. Special emphasis is noted on those grain boundaries with impurities, as discussed for LBPF [29,92]. von Mises stresses cause W cracking when thermally cycled at temperatures under its DBTT, supporting preheating and substrate heating being possible solutions to the cracking concerns in EBM-prepared W [19]. Materials built with a preheated powder bed of approximately 850 °C were found to have minor levels of cracking [90]. Suppressed crack formation in EBM W was reported when the build temperatures surpassed 1800 °C [93].

Substrate temperature and surface temperature were immensely influential on the presence of cracking in previous reports [3,6,19,94]. In LPBF, increasing the substrate temperature from 200 °C to 1000 °C significantly reduced cracking, with lower temperatures having causing the cracking of materials prepared using LPBF and EBM, respectively [3,90]. The use of metals, such as steel and titanium, as build substrates or build plates was also studied [24]. The spacing between the build plate and part with an EMB substrate was set to 1000 °C, and the elimination of cracking was demonstrated [29,91]. This was attributed to the theoretical model of mobile dislocations, alleviating cracking due to using a higher temperature than the DBTT of W.

Using higher substrate temperatures permits a lower temperature gradient during the heating and cooling cycle and leads to a reduction in the thermal stress imposed on the material as it cools. A support structure can be used to dissipate heat over a longer period and reduce the stress on materials further. The selection of substrate materials can also help to reduce cracks and the bond strength between the substrate and manufactured materials [24]. For example, Ti has been used to create a bond between the substrate build plate and the device due to its solubility with W. Crack density reduction was observed when the temperature was raised to 1500 °C to suppress cracking in W during EBM [26].

Nb-W alloys were shown to have similar crack propagation behavior as pure W and aided in suppressing crack severity [66]. Nickel (Ni) alloys were found to increase ductility and fracture toughness across all tested temperatures [55]. Alloying W with Mo improved the mechanical properties similar to Ni [95]. Y_2_O_3_ was found to have comparable results [96]. Vanadium (V) is another alloying element that can be used in EBM to improve the mechanical properties of W [94].

### 3.3. Microstructures of DED

Several studies have demonstrated the printability of W utilizing DED. These studies also revealed an obstacle in fully melting the W powder during deposition. Mixing between deposited W powder and the substrate material decreases the W content and leads to degradation of the desirable properties of W (i.e., hardness) [97]. Adding Fe and Cr in the melt pool is considered to increase the laser power due to deeper laser penetration into the steel substrate. Multilayer W prints were also evaluated. Intermetallic precipitates were observed and identified as an Fe-W phase. Iron-chromate phases were found. A significant compositional gradient was observed across the as-made component. The porosity observed within the W layers was likely due to gas trapped during solidification within the laser driven melt pools [97]. Such issues also have similar mitigating strategies in manipulating process parameters, as with other techniques [98].

Cracking can occur within the tracks of the laser and between the layers during DED, likely due to thermal stresses from the heating and cooling cycles created from the laser tracks. Cracking also was observed at the top of materials made using DED [97]. Cracks propagated down toward the substrate, causing W along the crack path to de-bond from the matrix and develop further [26,97]. Since DED-prepared W parts contain a large number of microcracks in particles near the top of deposits, remelting may influence the bottom materials, near the baseplate, when compared to the top [26]. However, it is worth noting that most DED setups do not have the laser power used in this particular study, and a lower laser power or fluence poses challenges in obtaining devices with desirable dimensions [97]. Several reports have shown success with W-Ni and W-Ni-Fe compositions to study the viability of DED to make W alloys [99].

**Figure 5:**
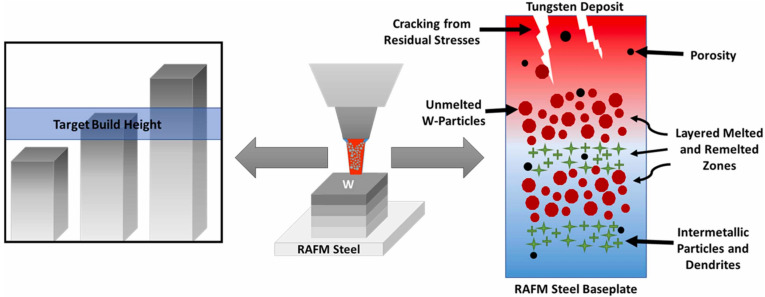
Challenges with the DED of W, including difficulty reaching build dimensions and cracking from stresses and porosity defects. Reprinted from Ref. [26] with permission; Copyright Elsevier 2022.

Several parameters, such as laser power, hatch spacing, and scan speed, can be modified to resolve these identified issues (see Figure 5). Substrate temperature may mitigate cracking through the alleviation of thermal stresses [94,100,101]. Substrate preheating could offer an improved melt pool morphology, with the increased melting of W particles [102]. Proper target dimensions can be achieved by adjusting machining processes such as the laser power, scan speed, powder feed rate, and powder quality [39,103]. Alterations in the substrate type and composition while maintaining the desired bonding level to the substrate itself could help to reduce unwanted intermetallic phases.

### 3.4. Thermal Properties and Performance

The thermal properties of W are consistent among the three techniques described within this review. Thermal conductivity is determined by the cracking behaviors and densification properties of the resultant product. Differences exist among the three methods, depending on the target densification, porosity, and cracking behaviors. The thermal conductivity of W prepared using LBPF was shown to be superior to SPS [104], and it was closer to that of ITER-grade W [58]. It was also correlated with the relative density using moderate scanning speeds [75]. Improvements by post processing treatments (i.e., hot isostatic pressing) also may eliminate voids and other defects [75].

An anisotropic relationship is observed in W made in LBPF. While the alloy content and cracking contribute to this behavior, the anisotropy of thermal diffusivity can be mitigated by the alloying of a high content of elements (i.e., Re) [81]. This implies that thermal diffusivity can be affected by the alloying element via isotropic scattering [81]. W materials manufactured via LPBF were shown to be capable of surviving intense thermal shocks in the JUDITH electron beam facility, where they did not experience extensive cracking or a loss of material [6].

Tungsten prepared in EBM was shown to have a similar thermal conductivity to standard W during surface plasma heat loading deterrence [105]. EBM was found to provide comparable W materials to traditional manufacturing in terms of plasma heat load exposure [105]. Also, EBM-prepared W had crack densities similar to those of standard W after plasma exposure. These findings suggest that EBM may offer a suitable avenue for PFC manufacturing, if coupled with crack reduction strategies [3,6,19,59].

## 4. Irradiation Effects of W as PFM

Irradiation occurs when a material is dosed with neutrons, ions, protons, or electrons. The striking atom is known as a primary knock-on atom (PKA) and can be approximated as elastic collisions. These PKAs will absorb the transferred energy and repeat the process with other atoms within the material. A displacement cascade occurs for the surrounding atoms, determined by the displacements per atom (dpa). The PKA can differentiate from the striking material and have varying energy levels upon bombardment. The bombarded material must contend with its own microstructure effects alongside these events. The segregation of interstitials and migration of impurity phases to sinks are examples if the displacement cascades impart enough energy.

The transmutation of species is also a possible outcome when altering the chemical makeup of the target. Furthermore, this environment is one in which any candidate material for fusion application must withstand scrutiny. The irradiation effects will be subjected to W, as a PFM, within the fusion environment, and specifically include neutron bombardment, transmutation effects, H retention (blistering), damage-causing void and loop formation, and impurity implantation. While it is well known that the accumulation of radiation damage in structural materials leads to the degradation of their physical and mechanical properties, little work has been conducted on the irradiation effects of AM W specifically. Instead, existing efforts have mostly been applied toward addressing concerns achieving those same properties using AM methods [2,81,106,107]. An analysis of the changes over time in the material can only be achieved postmortem or via a post-irradiation examination (PIE), rather than in situ or operando. Several options are available regarding radiation approaches. Ion irradiation can sometimes be substituted for neutron irradiation. However, equal dpa values from ion and neutron irradiations, respectively, do not necessarily describe the same effect. Neutron irradiation is at a higher dose rate. Thus, if ion substitution is selected, irradiation conditions that equate to the same irradiation of neutrons must be devised to serve as a good substitution [108]. The irradiation behavior of W is dependent on factors such as alloying elements, irradiation temperature, and dpa. For example, irradiation hardening behavior (obtained via fission reactor irradiation) found that the irradiation-induced hardening of W was dependent on the irradiation temperature, dpa, and alloy/impurity composition [109].

### 4.1. Neutron and Ion Irradiation Effects on W

**Figure 6:**
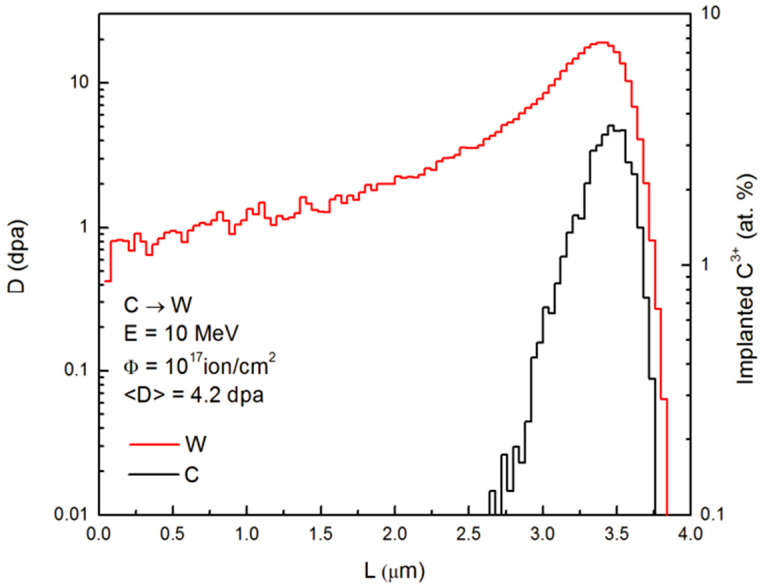
Distribution of primary defects and of the implanted carbon in the surface layer of W irradiated by carbon ions at 10 MeV. Φ is the fluence, <D> is the average dpa value, and E is the energy. Reprinted from Ref. [2] with permission; Copyright Elsevier 2015.

This neutron irradiation induces displacement damage throughout the W material as PFCs [110,111]. Figure 6 shows primary defect damage and the implanted C using the heavy ion irradiation of C_3_^+^ ions at 10 MeV with a fluence of 10^17^ ions/cm^2^ [2].

Displacement damage can produce a wide variety of damage geometries. Irradiated W has a short lifetime that is characteristic of the same dislocations. Intensities that were very close to the unirradiated samples indicated that the number of dislocations at the end of the irradiation was independent of microstructure. There was a second lifetime characteristic of large (>40) vacancy clusters at the temperature of 600 °C, in agreement with previous studies for all W grades [112]. These defects induce the embrittlement of W during irradiation. Low-temperature irradiation causes hardening, typical in BCC metals [8,113]. It has been reported that an increase in Vickers hardness and reduction in ductility are due to the transmutation of W, with Re- and Os-rich σ-precipitates [114].

Neutron testing in a fission reactor and ion irradiation are used to study neutron irradiation effects. Ion irradiation is expected to produce dense cascades similar to neutron irradiation [108,113]. However, transmutation does not occur during ion irradiation, unlike neutron irradiation. Thus, ion irradiation is limited in penetration depth and implants impurities into the material if self-ion irradiation is not used [108]. Ion irradiation, such as D, T, and He, will result in near-surface damage [115], contributing to blistering via retention [2].

The DBTT of W and W alloys was found to increase following neutron irradiation [116]. This change was attributed to a high density of voids and precipitates decreasing the dislocation velocity of the sample [117]. The DBTT can be used to summarize the effect of neutron irradiation on the mechanical properties of W. Increases in the DBTT depend on the irradiation temperature and fluence [8]. The irradiation of W offers a new perspective on the viability of possible alloying elements. Re can improve the workability and DBTT of W. Re was also shown to have worse embrittlement behaviors in irradiated W [8,109,118]. More recent studies reported that irradiation hardening was, in fact, suppressed by Re addition (<0.4 dpa). Significant hardening was observed at >1 dpa [109]. Other results reported a reduction in hardening up to 5 dpa [119]. The hardening behavior showed a function of Re addition before irradiation. The major defect clusters in pure W under these irradiation conditions were voids, with dislocation loops observed at lower dpa levels and at a much lower density [109].

### 4.2. Blistering

Neutron and ion irradiation bombardment results in damage both near the surface and in the bulk, which may lead to H retention and blistering behaviors during ion bombardment. Irradiation embrittlement is also a concern, especially with the neutron flux that W PFM will be exposed to. W has an absence of hydride formation. One of the effects of the bombardment of the energetic particles is the implantation of H isotopes and He into the material from plasma [24]. This leads to the formation of cavities, such as vacancies, grain boundaries, dislocations, and voids [111,120], which can lead to microcracking and eventually blistering [9]. These bubbles or blisters degrade the mechanical properties of the material, and they can burst and eject large amounts of gas and irradiated dust from the open cavity, leading to plasma instability [24,121].

Blistering can be produced by both H and He ion bombardment (voltage ranges from 1 eV to 10 keV or more) or by bombardment followed by plasma exposure [9]. Higher-energy ion bombardment is caused via α particle ejection due to the finite number of toroidal field (TF) coils [122]. Blistering is, thus, dominated by high-energy He ions. They can be trapped at vacancies in high-charge (Z) wall materials and cause cracks in bulk W, which is a concern for ITER [123,124]. Ion bombardment energies are less than a few hundred eV (relatively low) for the edge plasma conditions of other Tokamaks. When only H bombardment is a concern, He implantation and bubbles may still occur. [9,121,125,126,127,128]. Once the H atoms are implanted, they will diffuse into deeper regions and be trapped by pores, voids, or other sinks to become H bubbles, which leads to ion energy distribution [9]. Hydrogen bubbles cause internal stresses in the lattice, and they will eventually lead to microcracking and blistering. Additionally, the irradiated particulates can be expelled into the air when the blisters are opened, which is a safety concern during the PFC exchange process. The same is true with material instability due to high-charge Z release into the core plasma [121].

**Figure 7:**
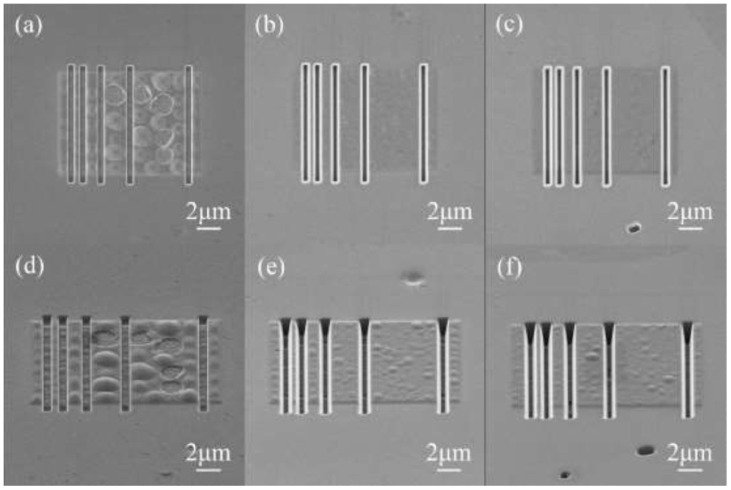
Tungsten surface after 30 keV, 1.0  × 10^22^ He^+^/m^2^ irradiation: (**a**–**c**) observations of blisters from the normal direction to the (001), (101), and (111) surfaces, respectively, and (**d**–**f**) blisters in tilting geometry (stage tilt 54°) corresponding to (**a**–**c**), respectively. Reprinted from Ref. [111] with permission; Copyright Elsevier 2020.

As the ion fluence increases, dust formation could become a concern. Blistering and impurity dust formation were observed for the Axially Symmetric Divertor Experiment (ASDEX) tokamak (fluence of 6 ∗ 10^25^ m^−2^) [129]. He-seeded D plasma was shown to reduce D retention and almost completely suppress blistering [9]. He implantation was reported to mitigate blistering due to the formation of nano-sized, high-density He bubbles near the surface, which might have served as a diffusion barrier to the bulk W [123]. This barrier of He bubbles was postulated to coalesce and make pores connected to the surface of the material as the fluence increased. Such phenomena could permit H to release and suppress the blistering phenomena [9]. A proposition is to use mechanical scratching to release trapped gas and reduce blistering behavior [128,130]. A “gas release channel” was used to decrease He blisters [111,131]. Nano grooves of a sufficiently small distance can reduce cracking, as shown in Figure 7.

Low-temperature pre-irradiation was shown to suppress blistering behavior [120]. Blistering was strongly temperature-dependent, and blister populations varied in population, distribution, sizes, and shapes at a higher temperature [120]. It is worth noting that much of the proton irradiation conditions have been low-energy irradiation (keV compared to MeV), in which the material is held at a lower irradiation temperature [10].

### 4.3. Transmutation

The absorption of neutrons is affected by nuclei during bombardment-induced transmutation, or changes in the mass number or atomic number of the original nuclide in the fusion environment [132]. Scattered instances of transmutation do not pose much of a threat to the properties of the bulk material. Transmutation elements can be found uniformly distributed or in clusters [133]. However, the accumulation of these transmutation products may cause detrimental effects to PFCs [132], such as irradiation hardening and brittleness [109]. These effects have been known to affect alloys of W more than pure W, depending on the level of irradiation [134].

Other effects may include alterations in recrystallization behavior and the stability of the W lattice [135]. Known transmutation products for pure W include Re, Ta, Os, and Hf [116,132]. W, Hf, Re, and Os were major products in order of decreasing concentration. Ta was detected in a recent study of W transmutation products [136]. These transmutation elements alter vacancy formation behaviors and they can affect properties such as the ductility and diffusion pathways of light gas elements [137]. The formation energy of these light gas elements was found to decrease due to the presence of transmutation elements, though their preferred site of formation was unaltered [137].

Minor products of Ir and Pt were also found over a 5-year irradiation timeframe [132]. W-Re alloys are not desirable due to their high burn-up levels and production of transmuted Os σ-phases. Therefore, the production of W as the primary transmutation product may serve to place Ta above Re. Re has been known to reduce swelling [132] and improve ductility. It could also be present in radiation-induced precipitates near grain boundaries [117,138].

Another kind of transmutation is the production of light gas elements, such as H and He. This occurs when a neutron enters a nucleus and a proton leaves simultaneously, also called an n, p reaction. Usually occurring at a high energy, these light transmutation products are common with the 14 MeV neutron irradiation present in fusion reactors. They are known to cause void swelling, although they are not produced in as great a quantity as heavier elements [109]. Thus, irradiated W is subject to a plethora of irradiation effects. Transmutation effects can possibly provide a mechanism to ensure the long-term stability (or instability) of W PFC with different alloying materials.

## 5. ML and Modeling of AM-Prepared Materials

It is often difficult and expensive to rely entirely on physical experiments to study neutron irradiation effects on PFMs. Computational methods are used to reduce the timeframe and cost. Modeling has been used for developing metals and alloys using AM. Prominent examples include ML (i.e., digital twin) and molecular dynamic (MD) simulations. These approaches are not limited to AM W only. Recent relevant modeling results are highlighted to assist future applications in W alloy manufacturing and material development.

The AM physical process or production system can be represented digitally [139,140]. Digital twins offer prediction of the resultant material properties via modeling. Digital twins can provide proactive alterations in steps to optimize the process parameters [140,141]. Digital twins are a prime resource in training models for a particular task, enabling ML-optimized data collection [141,142]. Such representation can overcome several issues in AM and allow for the optimization of instrument parameters, thereby improving part quality. Specifically, digital twins are an alternative to trial and error optimization approaches, and they mimic AM techniques to predict the part [139]. For example, alterations can be made for optimized outcomes by observing the process via sensors to monitor parameters of interest. Once a deviation occurs from a specified range, corrective actions are taken [140].

Digital twins were used in a microscopic-length-scale melt pool investigation and its sub processes. Parameters, such as spreading powder velocity distributions and evolving stresses within an AM part, can be studied [141,143,144]. Even thermal simulations can be combined with ML-paired digital twins to give information on the melt pool behavior during production [145]. In a recent study, an ML model was paired with a digital twin to study defect formation and user input adjustment before production. ML could aid in data cleaning, improve the accuracy of parameter applications to parts, and demonstrate the effectiveness of AM processes [146]. Since AM processing parameters and stress evolutions are a major concern in manufacturing W, digital twins can serve as a marvelous tool in modeling material behavior.

Digital twin models and ML algorithms have been applied specifically to nuclear applications. To bridge the gap between fast results and accurate simulations, a dynamic error database can be used with ML for autonomous calibration. Applications showed that physical quantities, such as temperature and pressure, can be modeled even during parallel runs, demonstrating the capacity for fast, accurate data collection in multiple simulations [147].

**Figure 8:**
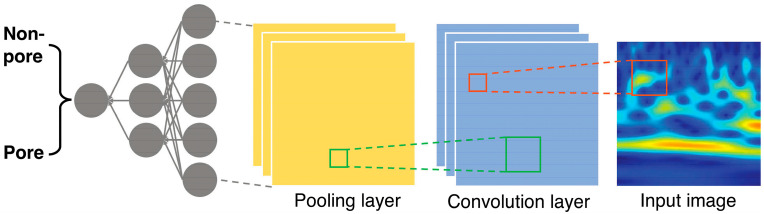
A diagram of a ML approach using sectioned scalograms as inputs and being classified as non-pores or pores to identify keyhole pore formation in the material. Reprinted from Ref. [148] with permission; Copyright American Association for the Advancement of Science 2023.

An ML model application to AM processes is depicted in Figure 8. A deep learning (DL) model was used for the identification of keyhole pore formation during manufacturing [148]. Each convolution layer sought to take information from the previous one, and down sampling was used to form a feature map for the manufactured parts. This map was then sampled to the pooling layers to reduce the parameter complexity. The final layer allowed for the classification of the input scalograms as “pore” or “non-pore”.

MD simulations are used to investigate the phase and composition of materials. Combining simulations and experiments can reduce the time and cost for materials’ design and development [149]. MD simulations can be used to model complex atomic interactions, such as the behavior of non-equilibrium melt pool physics and solidification processes in AM parts [150]. More specific investigations are possible, such as the melting behaviors of nano particulates and studies of particle shape [151,152]. MD simulations are utilized to understand the formation of defects, high-angle grain boundaries, or thermal stresses as functions of instrument parameters. The layer thickness effect can be investigated [153]. Similarly, MD has been used to study the influences of preheating temperature on melt pool behavior and the thermal properties [150]. MD simulations were used to study the phase transitions during AM processes, thermal cyclical transformations, and thermal history [154].

ML advances are seeing increased applications in MD simulations. For example, TorchMD provides a deep neural network and biomolecular dynamics simulations in protein folding [155]. Multiple types of emerging applications of ML for MD simulations were recently summarized, highlighting neural networks and generative network approaches for kinetics and thermodynamics [156]. This approach is applicable to the AM development of materials. For instance, ML was employed with a Markov state model to study AM Al-Ti systems and to assist in the observation of diffusion mechanisms during printing, offering a promising application in metal alloys [157].

ML applications in the AM of tungsten and alloys are not limited to MD simulations and digital twin models. Simulations of melt pool behavior are of interest in AM due to their direct correlation with the behaviors of fabricated components [158]. Ren et al. employed a two-stage ML model to predict melt pool behavior during AM scanning [159]. Previous methods for simulating this behavior include finite element analysis (FEA) modeling and fluid mechanics; however, they are problematic due to high-power infrastructure requirements for proper utilization [160]. ML was used to properly predict melt pool size as a function of the AM process parameters [159]. Similar results were obtained when employing a neural network model to predict the temperature and melt pool fluid dynamics in metal AM processes [161]. The efforts showed ML’s capability to accurately predict desired behaviors [161]. ML was also used to understand the powder spreading for metal-based AM [162]. A framework was presented based on discrete element methods to predict and characterize powder bed behaviors. These predictions were used to produce a process map that can be used to produce components at a desired roughness, using the spreading behavior to enhance the desired surface properties [162].

## 6. Gaps and Recommendations

W and W alloys are difficult to print due to high melting temperatures and their inherent brittleness. However, progress in AM processes has shown a promising printability of materials. Cracking continues to be the primary challenge to overcome. EBM shows promise in creating crack-free samples and strategies. Novel alloy development is an important strategy in AM. Microstructural control has been demonstrated for other metallic materials. However, it has not been fully demonstrated for AM-based W [65,144,163,164]. Porosity is less investigated. Mechanical properties at elevated temperatures are especially limited regarding practical applications in PFCs. Resolving these issues would provide great benefits toward improving the capacity for the creation of the large and complex parts needed for PFCs.

Microstructural control, especially over grain development, is known to reduce thermal stress. The latter is a main cause of cracking for AM W [65,163,164]. The addition of nanoparticles could improve crack suppression through the reduction in harmful impurities like O. Further studies in combination with higher build temperatures would be appealing [77]. A comprehensive study of cooling rates would go great lengths to further the understanding of crack suppression. Substrate heating is a strategy for EBM. Though gaining traction in LPBF, less is known. Information is scarcer regarding DED [3].

The mechanical testing of AM-prepared W has been performed. However, comprehensive results encompassing various mechanical properties with different methods are missing. A wide range of instrument parameters can produce a variety of materials. Testing can be performed in a wide range of settings. Optimizing the thermal performance to be closer to ITER-grade W would improve the thermal properties of AM-prepared W materials. This is a subject that would benefit from additional exploration. Although many studies have put forth hypotheses on the origin of brittleness, comprehensive studies of impurities, such as O and H effects on brittleness and properties, are missing. Optimizing parameters such as the preheating conditions and cooling rate would be of interest in improving the understanding of their influences on cracking behavior. Moreover, efforts toward combining these processes for a reduction in cracking would realize the adoption of AM for W-based PFC production.

Another possibility for future efforts is focusing on the upscaling of laboratory-scale AM methods towards larger industrial-scale applications. If such techniques are to be used to feed the requirements for a fusion fleet, consistent success should be sought accordingly to ensure that laboratory-scale production strategies are achievable, Specific applications that could be explored would be raising concerns and issues that arise in the cracking behavior of AM-prepared alloys at an industrial scale. Another challenge is the consistency of mechanical and microstructural behaviors at large scales and the feasibility of maintaining consistent parameters while allowing for alterations as needed. The utilization of ML may help in the scaling up of such strategies when employed effectively.

ML has garnered much attention in recent years and permeated many scientific disciplines due to its ability to bridge physical observations with simulations. ML models can potentially play a critical role in the AM development of W-based materials in multiple areas, including parameter optimization, the modeling of complex processes, quality control, and energy management. The expansion of simulated manufacturing methods would enable cost reduction and effective processes for material evaluation without costly physical testing. These processes could assess the entire building concept, workflows, and devices. Such simulations would enable the near real-time or real-time assessment of AM materials during manufacturing or inclusion within a working environment. These strategies may help future AM studies via the in situ monitoring of defects and investigation of the effects of in-process alterations in parameters.

One specific area for further investigation is ML modeling efficiency. Digital twins must be entrusted to handle the real-time monitoring and control of large and complex physical data. Therefore, simulations which contain multiscale and multidisciplinary interactions and the capacity for fast, approachable adoption have yet to be fully realized. Several strategies towards this end have been proposed, for instance, utilizing surrogate models to reduce large quantities of data from the data science and engineering point of view. Establishing a dynamic framework in ML can ensure consistent productization at large scales. Even if variations in build parameters are required, such frameworks would facilitate rapid alterations in situ.

Irradiation effects on W and W alloys present a challenge for AM technologies. Research that combines the knowledge of transmutation in relation to the ratios of alloying elements can facilitate the design and development of suitable PFMs. However, there is a lack of comprehensive studies on the irradiation effects of AM-prepared W. This gap of potentially prominent alloy candidates and their responses to neutron irradiation needs closing.

The transmutation effects that W PFCs are exposed to in fusion power plants provide a unique challenge. An element included in a W alloy will be subject to transmutation effects as well. If an element successfully mitigates some problems during manufacturing, yet produces harmful transmutation products, then this might nullify that alloy composition. Due to the lack of comprehensive data on subjecting AM W parts to irradiation effects, one pathway would be to identify and study the behavior of an alloy comprising W and elements whose primary transmutation product is W. This theoretical composition might be able to transition to a greater-percentage pure W over its lifetime, which, depending on the desired part lifetime, might prove beneficial.

## 7. Conclusions

The utilization of AM-prepared W and alloys as PFMs is a field with rapid growth for developing fusion suitable devices. This review provides an overview of recent efforts to bring forth reliable parts with suitable mechanical properties for applications in fusion power plants. We chose to focus on LPBF, DED, and EBM because they hold high promise for W alloy development. Updates on these three methods relevant to W manufacturing are provided. The most outstanding issue among these techniques is the cracking of the produced parts. Other problems include impurities and raw material characteristics, which worsen cracking. Strategies have been developed, such as preheating and parameter optimization, that result in a reduction in or elimination of defects. Grain refinement and alloying offer additional options.

The irradiation effects of W are discussed. The displacement damage and blistering effects of traditionally manufactured W are identified. The transmutation effects of PFCs present a unique challenge. Elements included in a W alloy will be subject to transmutation. The choice of alloying is a topic of vigorous ongoing research. Future work and directions for the AM of W include efforts toward parameter refinement. A comprehensive study across multiple parameter spaces for a particular technique would be valuable in demonstrating a range that produces successful printings.

There are a lack of data on subjecting AM-prepared W parts to irradiation effects. An investigation of these effects on AM W parts would be beneficial. Future efforts are also needed in studying the transmutation behaviors of promising alloy compositions to determine if any harmful products are produced. An interesting topic would be to evaluate primary transmutation products to address the need for and realization of W and alloys as PFMs.

## Data Availability

No new data were created or analyzed in this study. Data sharing is not applicable to this article.

## Supplementary Materials

The following supporting information can be downloaded at: https://www.mdpi.com/article/10.3390/ma17092104/s1, Figure S1: A W-Cr phase diagram. with permission; Copyright Elsevier 2016; Figure S2: Complex AM W lattice structure samples. Reprinted from Ref. [6]. with permission; Copyright Elsevier 2022; Figure S3: Observed states of pure tungsten fabricated through EBM using different parameters. (a) Limited fusion, (b) insufficient fusion, (c) proper fusion, and (d) excessive fusion. Reprinted from Ref. [90]. with permission; Copyright Elsevier 2019; Figure S4: Electron backscatter diffraction (EBSD) images of two sides of a L-PBF W sample showing surface cracks. (a,c) As-printed W cross sections on different axes. Longitudinal, branched, and parallel build direction (BD) cracks are visible (red and blue arrows). (b,d) EBSD inverse pole figure (IPF) maps of (a,c) showing ladder-shaped grains, cracks along grain boundaries and laser tracks. Reprinted from Ref. [88]. with permission; Copyright Elsevier 2018; Table S1: A list of suppliers and powder types used in Ref [3]; Table S2: Operating ranges of Laser powder bed fusion, direct energy deposition, electron beam melting, and wire arc additive manufacturing. Adapted from Ref. [26].

## Glossary

AI	Artificial Intelligence
AM	Additive Manufacturing
BCC	Body-Centered Cubic
D	Deuterium
DBTT	Ductile to Brittle Transition Temperature
DED	Direct Energy Deposition
DEMO	Demonstration Power Plant
DOE	Department of Energy
DL	Deep Learning
dpa	displacements per atom
EDS	Energy-Dispersive X-ray Spectroscopy
EBM	Electron Beam Melting
EBSD	Electron Backscatter Diffraction
Fe	Iron
FEA	finite element analysis
Hf	Hafnium
He	Helium
H	Hydrogen
HCP	Hexagonal Close-Packed
IPF	Inverse Pole Figure
ITER	International Thermonuclear Experimental Reactor
KAM	Kernel Average Misorientation
LPBF	Laser Powder Bed Fusion
Li	Lithium
ML	Machine Learning
MD	Molecular Dynamics
Mo	Molybdenum
Nb	Niobium
N	Nitrogen
Ni	Nickle
Os	Osmium
O	Oxygen
P	Phosphorus
PALS	Positron Annihilation Lifetime Spectroscopy
PFC	Plasma-Facing Components
PFM	Plasma-Facing Materials
PKA	Primary Knock-On Atoms
PSD	Particle Size Distribution
Re	Rhenium
Ru	Ruthenium
SPS	Spark Plasma Sintering
Ta	Tantalum
TF	Toroidal Field
Tc	Technetium
Ti	Titanium
T	Tritium
V	Vanadium
W	Tungsten
XPS	Zirconium
Z	Nuclear Charge Number
Zr	Zirconium
