# Supplementary material for: Progress and Challenges of Additive Manufacturing of Tungsten and Alloys as Plasma-Facing Materials
Source: Materials (Basel). 2024 Apr 29;17(9):2104. doi: 10.3390/ma17092104 (PMC11084790; doi:10.3390/ma17092104)
Supplement: Supplementary file 1 [file materials-17-02104-s001.zip › materials-2965634-supplementary.pdf]

materials

Supporting Information

Progress and Challenges of Additive Manufacturing of Tungsten and Alloys as Plasma Facing Materials

Logan Howard [1,2], Gabriel D. Parker [1] and Xiao-Ying Yu [1,2,*]

[1] Materials Science and Technology Division, Oak Ridge National Laboratory, Oak Ridge, TN 37830, USA
[2] The Bredesen Center, 310 Ferris Hall 1508, Middle Dr Knoxville, TN 37996, USA
* Correspondence: Corresponding Author: E-mail: yuxiaoying@ornl.gov; Tel: 865-574-4628

Contents

Supplemental Tables S-3

Table S1. A list of suppliers and powder types used in Ref [3].. S-3

Table S2. Operating ranges of Laser powder bed fusion, direct energy deposition, electron beam melting, and wire arc additive manufacturing. Adapted from Ref. 26. .... S-4

Supplemental Figures.. S-5

Figure S1. A W-Cr phase diagram [179]. Reprinted from Calvo, A. Manufacturing and testing of self-passivating tungsten alloys of different composition. Nuclear Materials and Energy, 2016. 9: p. 422-429. Creative commons attribution, no alterations made. S-5

Figure S2. Complex AM W lattice structure samples. Reprinted from Müller, A.V. Müller, D. Dorow-Gerspach, M. Balden, M. Binder, B. Buschmann, B. Curzadd, T. Loewenhoff, R. Neu, G. Schlick, J.H. You, Progress in additive manufacturing of pure tungsten for plasma-facing component applications, Journal of Nuclear Materials, 2022. Creative commons attribution, no alterations made........................ S-6

Figure S3. Observed states of pure tungsten fabricated through EBM using different parameters. (a) Limited fusion, (b) insufficient fusion, (c) proper fusion, and (d) excessive fusion. Reprinted from G. Yang, P. Yang, K. Yang, N. Liu, L. Jia, J. Wang, H. Tang, Effect of processing parameters on the density, microstructure and strength of pure tungsten fabricated by selective electron beam melting, International Journal of Refractory Metals and Hard Materials, 84 (2019) 105040-105040.. S-7

Figure S4. Electron backscatter diffraction (EBSD) images of two sides of a L-PBF W sample showing surface cracks. (a, c) As-printed W cross sections on different axes. Longitudinal, branched, and parallel build direction (BD) cracks are visible (red and blue arrows). (b, d) EBSD inverse pole figure (IPF) maps of (a, c) showing ladder-shaped grains, cracks along grain boundaries and laser tracks. Reprinted from Wang, D.; Yu, C.; Zhou, X.; Ma, J.; Liu, W.; Shen, Z. Dense Pure Tungsten Fabricated by Selective Laser Melting. *Applied Sciences 2017, Vol. 7, Page 430* 2017. Creative commons attribution, no alterations made. S-8

References.. S-9

Supplemental Tables

Table S1. A list of suppliers and powder types used in Ref [3].

Powder type	Supplier
Spheroidised 225 W powder, W -25	Tekna Advanced Materials Inc.
Partially spheroidised W powder	Global Tungsten & Powders Corp
Polygonal W powder, HC 4000	H. C. Starck GmbH,

Table S2. Operating ranges of Laser powder bed fusion, direct energy deposition, electron beam melting, and wire arc additive manufacturing. Adapted from Ref. [26].

	LPBF	DED	EBM	References
**Particle size (μm)**	5–50	50–150	20–105	[165,166]
**Source Power (W)**	10^2–10^3	10^2–10^4	10^2–10^3	[26,167]
**Cooling rate (K/s)**	10^5–10^7	10^2–10^5	10^3–10^4	[26,168]
**Temperature gradient (K/m)**	10^6–10^7	10^4–10^6	10^4–10^5	[26,169,170]
**Beam Size (μm)**	30–200	10^2–10^3	10^2–10^3	[26,171,172,173]
**Scanning speed (mm/s)**	10–10^3	10–10^2	10–10^3	[26,174,175]
**Environment**	Ar, N_2	Ar	Vacuum, trace He	[26,176,177]
**Spattering**	Yes	No	No	[26,178]
**Material Waste**	High	Minimal	High	[26]
**Pre-Sintering**	No	No	Yes	[26]
**Pre heat temperature (K)**	0–1273	0–773	0–1528	[3,101,136]

Supplemental Figures

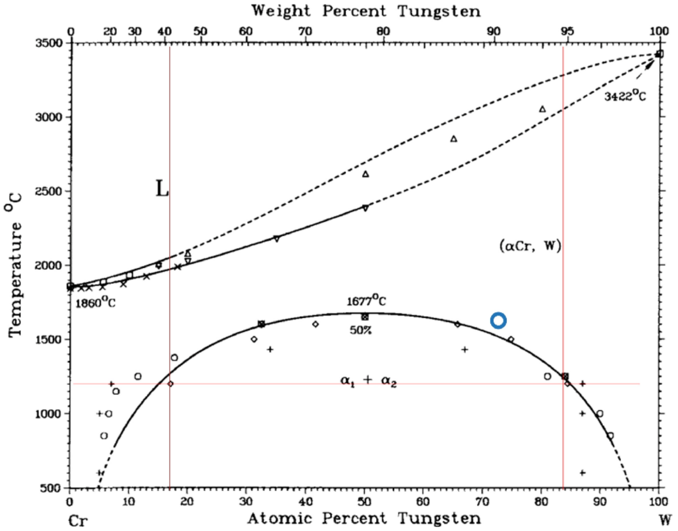

Figure S1. A W-Cr phase diagram [179]. Reprinted from Ref. [179]. with permission; Copyright Elsevier 2016.

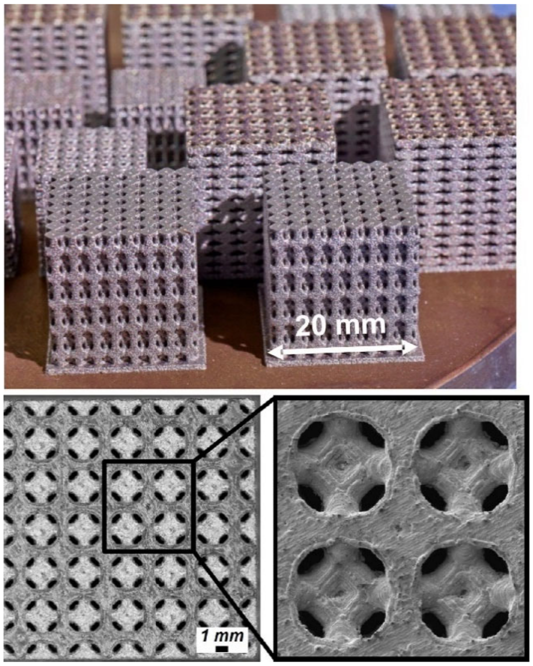

Figure S2. Complex AM W lattice structure samples. Reprinted from Ref. [6]. with permission; Copyright Elsevier 2022.

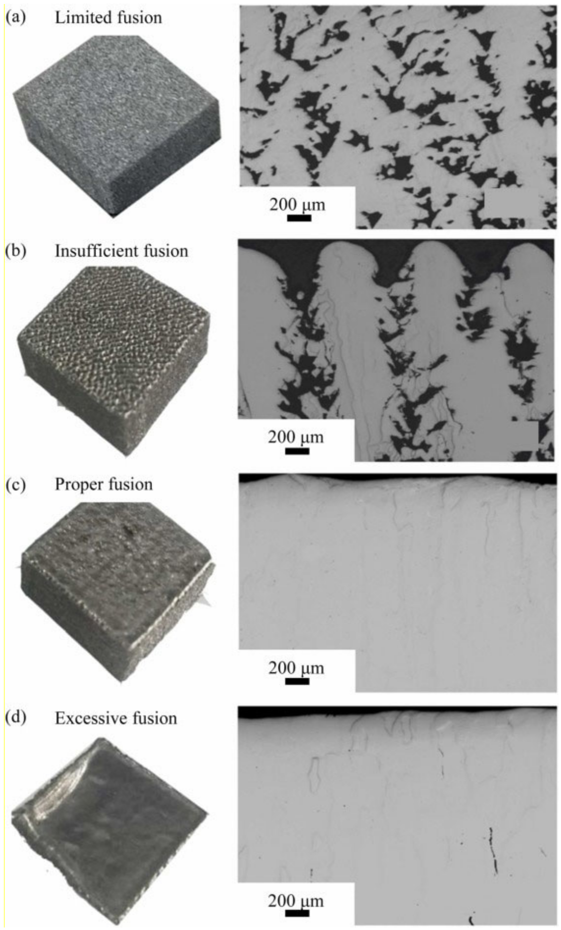

Figure S3. Observed states of pure tungsten fabricated through EBM using different parameters. (**a**) Limited fusion, (**b**) insufficient fusion, (**c**) proper fusion, and (**d**) excessive fusion. Reprinted from Ref. [90]. with permission; Copyright Elsevier 2019.

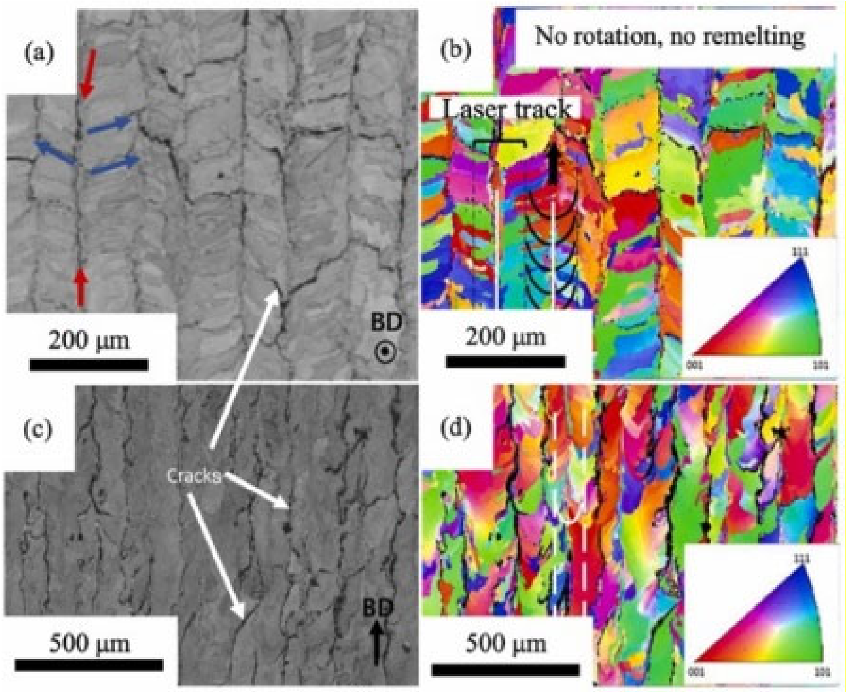

Figure S4. Electron backscatter diffraction (EBSD) images of two sides of a L-PBF W sample showing surface cracks. (**a, c**) As-printed W cross sections on different axes. Longitudinal, branched, and parallel build direction (BD) cracks are visible (red and blue arrows). (**b, d**) EBSD inverse pole figure (IPF) maps of (a, c) showing ladder-shaped grains, cracks along grain boundaries and laser tracks. Reprinted from Ref. [88]. with permission; Copyright Elsevier 2018.

References

3. Müller, A.V.; Schlick, G.; Neu, R.; Anstätt, C.; Klimkait, T.; Lee, J.; Pascher, B.; Schmitt, M.; Seidel, C. Additive manufacturing of pure tungsten by means of selective laser beam melting with substrate preheating temperatures up to 1000 °C. Nucl. Mater. Energy **2019**, *19*, 184–188.

6. Müller, A.V.; Dorow-Gerspach, D.; Balden, M.; Binder, M.; Buschmann, B.; Curzadd, B.; Loewenhoff, T.; Neu, R.; Schlick, G.; You, J.H. Progress in additive manufacturing of pure tungsten for plasma-facing component applications. Journal of Nuclear Materials **2022**, *566*, 153760.

26. Talignani, A.; Seede, R.; Whitt, A.; Zheng, S.; Ye, J.; Karaman, I.; Kirka, M.M.; Katoh, Y.; Wang, Y.M. A review on additive manufacturing of refractory tungsten and tungsten alloys. Addit. Manuf. **2022**, *58*, 103009.

35. Wang, D.; Yu, C.; Zhou, X.; Ma, J.; Liu, W.; Shen, Z. Dense Pure Tungsten Fabricated by Selective Laser Melting. Appl. Sci. **2017**, *7*, 430.

88. Wang, D.Z.; Li, K.L.; Yu, C.F.; Ma, J.; Liu, W.; Shen, Z.J. Cracking Behavior in Additively Manufactured Pure Tungsten. Acta Metall. Sin. (Engl. Lett.) **2019**, *32*, 127–135.

90. Yang, G.; Yang, P.; Yang, K.; Liu, N.; Jia, L.; Wang, J.; Tang, H. Effect of processing parameters on the density, microstructure and strength of pure tungsten fabricated by selective electron beam melting. Int. J. Refract. Met. Hard Mater. **2019**, *84*, 105040.

101. Fallah, V.; Alimardani, M.; Corbin, S.F.; Khajepour, A. Impact of localized surface preheating on the microstructure and crack formation in laser direct deposition of Stellite 1 on AISI 4340 steel. Appl. Surf. Sci. **2010**, *257*, 1716–1723.

136. Drescher, P.; Sarhan, M.; Seitz, H. An Investigation of Sintering Parameters on Titanium Powder for Electron Beam Melting Processing Optimization. Materials **2016**, *9*, 974.

165. Vock, S., Kloden, B.;Kirchner, A.; Weißgärber,T.; Kieback, B. Powders for powder bed fusion: a review. Prog. Addit. Manuf. **2019**, *4*, 383–397.

166. Seyam, M.S., P. Koshy, and M.A. Elbestawi, Laser Powder Bed Fusion of Unalloyed Tungsten: A Review of Process, Structure, and Properties Relationships. Metals, **2022**, *12(2)*, 274.

167. Cai, C. and K. Zhou, Chapter 7 - Metal additive manufacturing, in Digital Manufacturing, C.D. Patel and C.-H. Chen, Editors. 2022, Elsevier. p. 247–298.

168. Lass, E.A.; Stoudt, M.R.; Williams, M.E.; Katz, M.B.; Levine, L.E.; Phan, T.Q.; Gnaeupel-Herold, T.H. and Ng, D.S. Formation of the Ni3Nb δ-Phase in Stress-Relieved Inconel 625 Produced via Laser Powder-Bed Fusion Additive Manufacturing. Metall. Mater. Trans. A, **2017**, *48(11)*, 5547–5558.

169. Hooper, P.A., Melt pool temperature and cooling rates in laser powder bed fusion. Addit. Manuf. **2018**, *22*, 548–559.

170. Svetlizky, D.; Das, M.; Zheng, B.; Vyatskikh, A.L.; Bose, S.; Bandyopadhyay, A.; Schoenung, J.M.; Lavernia, E.J.; Eliaz, N. Directed energy deposition (DED) additive manufacturing: Physical characteristics, defects, challenges and applications. Mater. Today, **2021**, *49*, 271–295.

171. Sow, M.C.; De Terris, T.; Castelnau, O.; Hamouche, Z.; Coste, F.; Fabbro, R.; Peyre, P. Influence of beam diameter on Laser Powder Bed Fusion (L-PBF) process. Addit. Manuf. **2020**, *36*, 101532.

172. Zhao, C.; Fezzaa, K.; Cunningham, R.W.; Wen, H.; De Carlo, F.; Chen, L.; Rollett, A.D.; Sun, T. Real-time monitoring of laser powder bed fusion process using high-speed X-ray imaging and diffraction. Sci. Rep. **2017**,*7(1),* 3602.

173. Kong, Y.; Zhao, L.; Zhu, L.; Huang, H. The selection of laser beam diameter in directed energy deposition of austenitic stainless steel: Accomprehensive assessment. Addit. Manuf. **2022**, *52*, 102646.
174. Chen, Z.; Lu, Y.; Luo, F.; Zhang, S.; Wei, P.; Yao, S.; Wang, Y. Effect of Laser Scanning Speed on the Microstructure and Mechanical Properties of Laser-Powder-Bed-Fused K418 Nickel-Based Alloy. Materials (Basel), **2022**,*15(9),* 3045.
175. Kirchner, A.; Klöden, B.; Luft, J.; Weißgärber, T.; Kieback, B. Process Window for Electron Beam Melting of Ti-6Al-4V. Powder Metall. **2015**, *58(4)*, 246–249.
176. Amano, H.; Ishimoto, T.; Suganuma, R.; Aiba, K.; Sun, S.H.; Ozasa, R.; Nakano, T. Effect of a helium gas atmosphere on the mechanical properties of Ti-6Al-4V alloy built with laser powder bed fusion: A comparative study with argon gas. Addit. Manuf. **2021**, *48*, 102444.
177. Singh, S.; Sharma, V.S.; Sachdeva, A.; Sharma, V.; Kaur, D.; Isanaka, B.R.; Kushvaha, V. Processing and Manufacturing Ti6Al4V-Based Structures and Composites Using SLM and EBM: A Review, in Additive and Subtractive Manufacturing of Composites, S. Mavinkere Rangappa, et al., Editors. 2021, Springer Singapore: Singapore. p. 73–103.
178. Young, Z.A.; Guo, Q.; Parab, N.D.; Zhao, C.; Qu, M.; Escano, L.I.; Fezzaa, K.; Everhart, W.; Sun, T.; Chen, L. Types of spatter and their features and formation mechanisms in laser powder bed fusion additive manufacturing process. Addit. Manuf. **2020**, *36*, 101438.
179. Calvo, A.; García-Rosales, C.; Koch, F.; Ordás, N.; Iturriza, I.; Greuner, H.; Pintsuk, G.; Sarbu, C. Manufacturing and testing of self-passivating tungsten alloys of different composition. Nucl. Mater. Energy, **2016**, *9*, 422–429.
